# Spontaneous transverse colon volvulus in a patient with Duchenne muscular dystrophy: An unreported complication

**DOI:** 10.1016/j.radcr.2023.03.027

**Published:** 2023-04-12

**Authors:** Pietro Pitrone, Antonino Cattafi, Francesca Magnani, Maria Ludovica Carerj, Italo Giuseppe Bellone, Giuseppe Nirta, Enrico Monsù, Dora Bonanno, Renato Trimarchi, Alessandro La Face, Maria Adele Marino, Carmelo Sofia

**Affiliations:** a Section of Radiological Sciences, Department of Biomedical Sciences and Morphological and Functional Imaging, University of Messina, Policlinico "G. Martino" Via Consolare Valeria 1, 98100, Messina, Italy; b Department of Radiodiagnostic, Oncologic Radiotherapy and Ematology, Università Cattolica del Sacro Cuore, Fondazione Policlinico Universitario A. Gemelli IRCCS, L.go A. F.Vito 1 Gemelli 8, 00168, Rome, Italy

**Keywords:** Duchenne muscular dystrophy, Gastrointestinal, Chronic constipation, Transverse colon volvulus

## Abstract

A 22-year-old male patient having Duchenne muscular dystrophy (DMD) and chronic constipation referred to the emergency department of our hospital for severe abdominal pain and signs of bowel obstruction. Contrast-enhanced computed tomography of the abdomen demonstrated mechanical ileus due to a volvulus of the transverse colon. Torsion of the transverse mesocolon and multiple ischemic areas with focal intestinal wall perforations were confirmed during surgical exploration and a subtotal colectomy was performed. DMD is frequently associated to gastrointestinal motility disorders, including chronic constipation, and life-threatening conditions like intestinal pseudo-obstruction and sigmoid volvulus. To date to our knowledge, transverse colon volvulus represents an unreported condition among patients with DMD.

## Introduction

Duchenne muscular dystrophy (DMD) is the most common inherited neuromuscular disorder in children. It is associated to mutations in the gene coding for dystrophin protein and leads to progressive muscular weakness and disability since a very young age [1], [2], [3], followed by a severe cardio-respiratory failure between the second and the third decade of life [4], [5], [6]. Other minor symptoms involve the urinary and, most importantly, the gastrointestinal tract, including motility disorders (gastric distension, chronic constipation and diarrhea) as well as life-threatening conditions like intestinal pseudo-obstruction and volvulus of the sigmoid colon [6], [7], [8], [9], [10].

## Case report

**Fig. 1:**
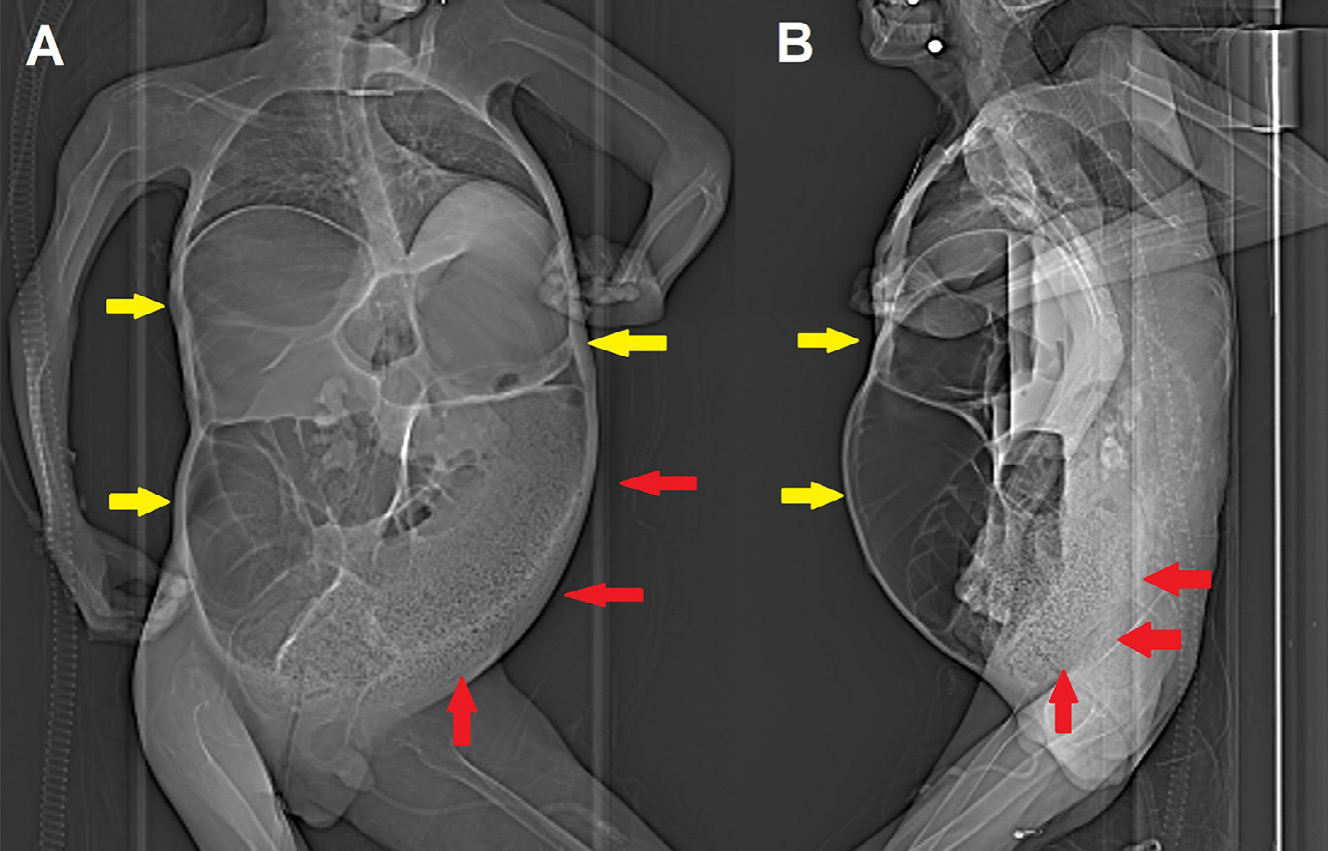
Anteroposterior (A) and lateral (B) scout images obtained before contrast-enhanced CT already show extensive bowel dilatation (yellow arrows) and left colon fecaloma (red arrows).

**Fig. 2:**
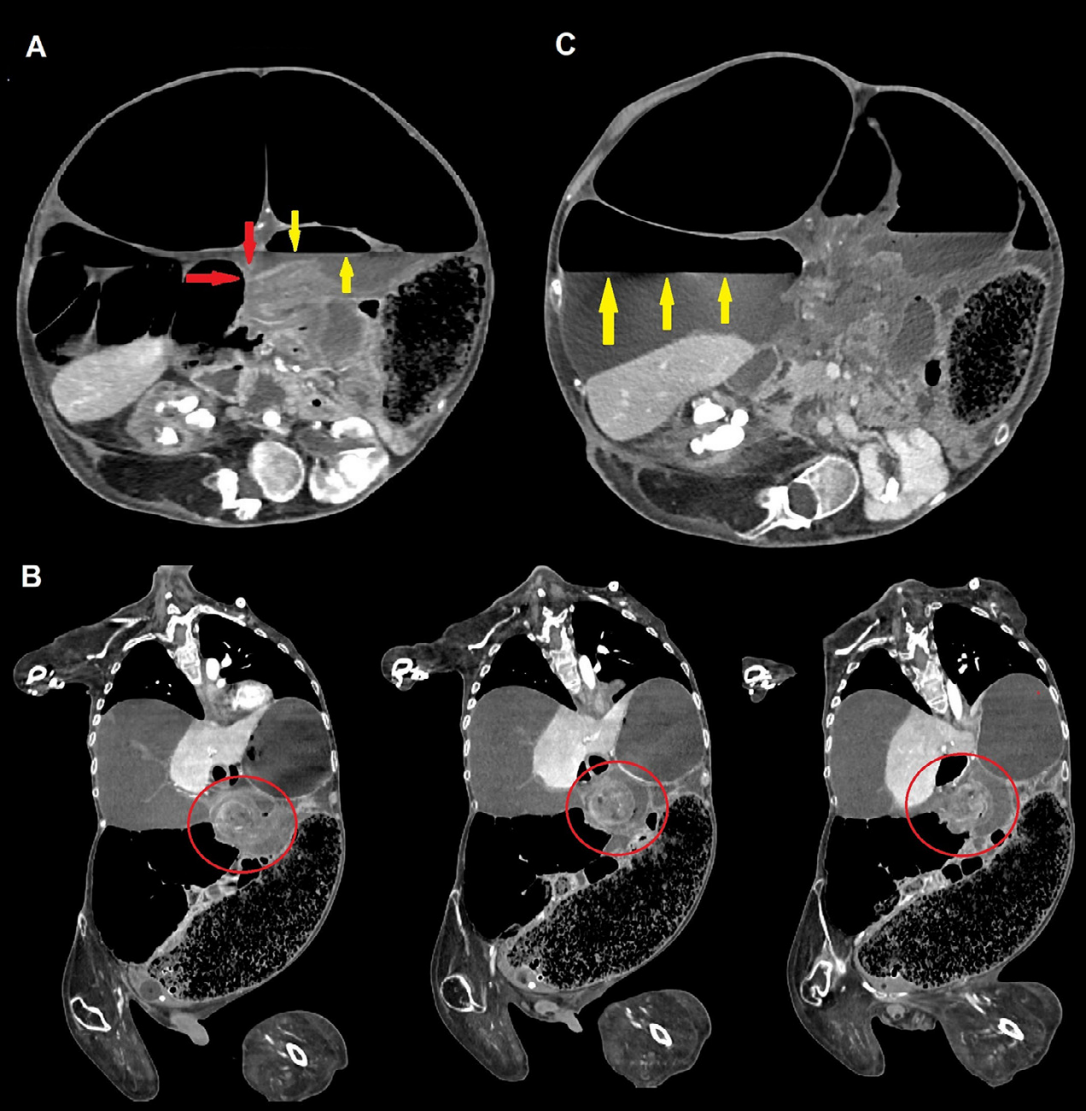
Contrast-enhanced CT scans of the abdomen demonstrating volvulus of the transverse colon. Axial scans (A-C) show an abrupt caliber change (“beak sign,” red arrows, commonly seen in many types of mechanical ileum) representing the site of the occlusion, and proximal air-fluid level (yellow arrows). Coronal view (B), obtained with multiplanar reconstructions (MPR), demonstrates the same loop twisting around the long axis of its meso (“whirl sign,” red circles); most of the right colon is displaced medially, as well as jejunal loops, due to the partial involvement of the root of the mesentery. Significant right colic dilatation with atony is present; the caliber of the caecum measures more than 17 cm and an evident air-fluid level is identified (yellow arrows).

**Fig. 3:**
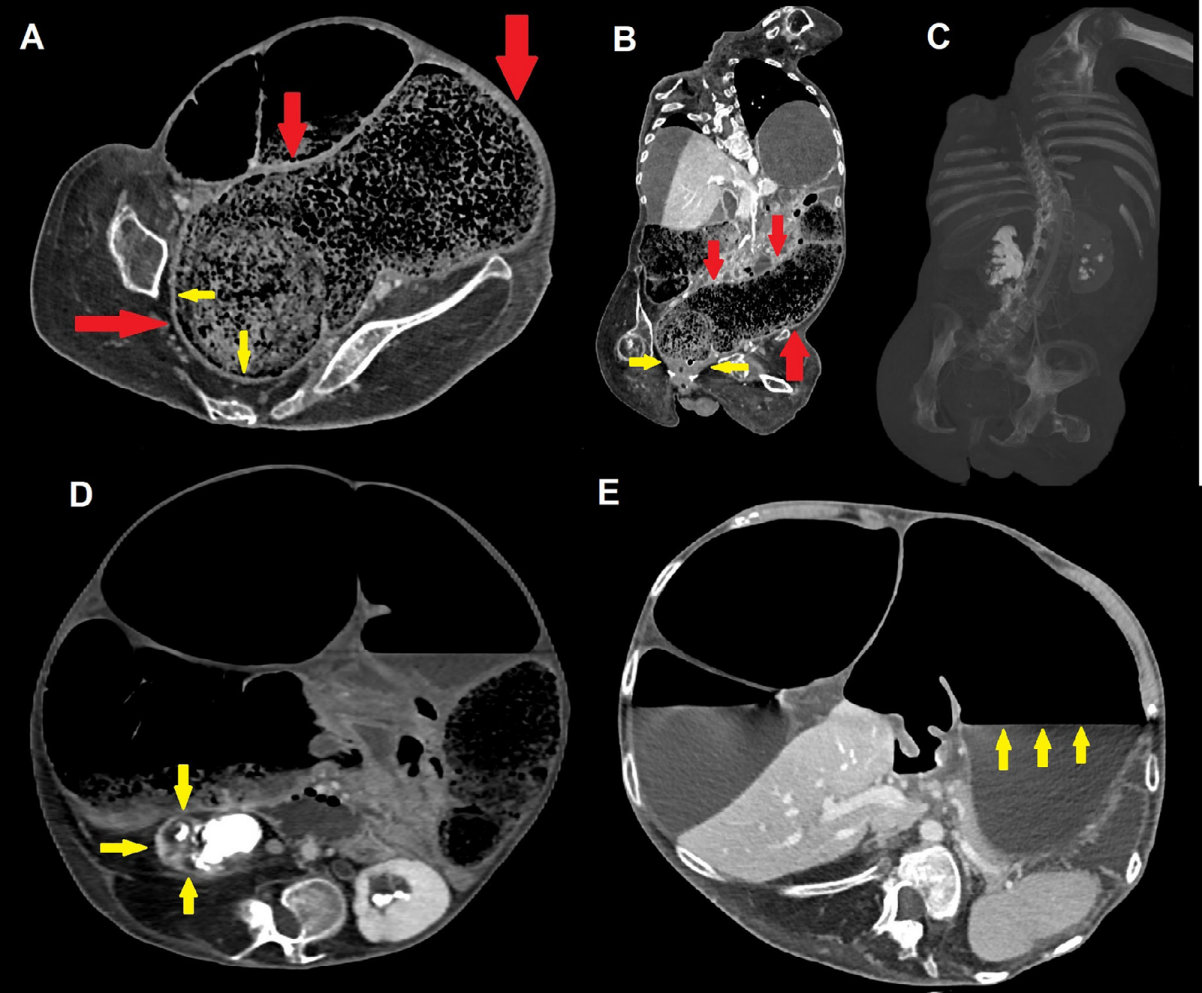
Axial (A, D-E) and coronal (B, C) contrast-enhanced CT scans demonstrate an extensive recto-sigmoid fecaloma measuring more than 30 × 10 cm (red arrows); chronic thickening of rectal walls is visible (yellow arrows), as well as compressive bilateral grade III hydronephrosis with staghorn stones and thinning of renal parenchyma, frequently seen in DMD patients (C and D, yellow arrows). Dilatation with atonia and an evident air-fluid level also involves the stomach (E, yellow arrows), further witnessing the multifocal nature of gastrointestinal motility disorders in patients with DMD.

**Fig. 4:**
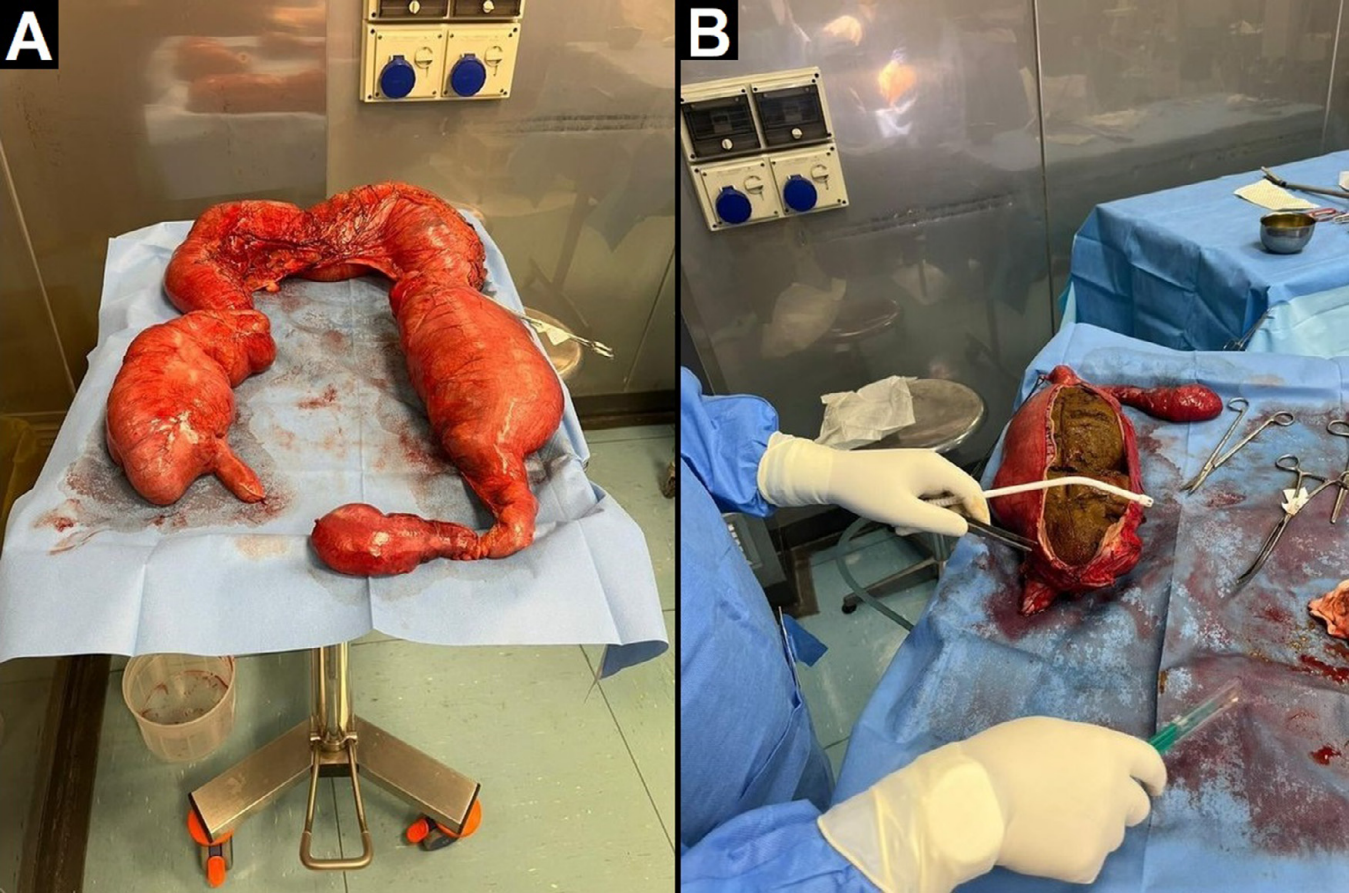
Pictures from the operating room showing subtotal colectomy. Multiple ischemic areas are visible, especially at the level of the transverse colon, where focal perforation with initial fecal leakage is present. There is also distension of the caecum and descending colon (A), the latter containing a huge fecaloma (B).

A 22-year-old male patient having DMD, cardio-pulmonary insufficiency requiring non-invasive ventilation, renal failure, and a history of constipation managed with Movicol and other prokinetics, referred to the emergency department of our hospital complaining severe nausea and abdominal pain lasting 2 days. The clinical evaluation showed a rounded and tender abdomen associated to signs of bowel obstruction. Contrast-enhanced computed tomography of the abdomen (scout images in Fig. 1) demonstrated a mechanical ileus caused by a volvulus of the transverse colon (Fig. 2); an extensive fecaloma occupying the lower third of the descending colon and the recto-sigmoid colon was seen (Fig. 3). The patient thus required an emergency surgical exploration: a diffuse megacolon with atony and multiple ischemic areas came out, especially within the transverse colon where were identified focal perforations and an initial fecal leakage. Complete torsion of the transverse mesocolon was also confirmed and consequently a subtotal colectomy was performed (Fig. 4).

## Discussion

DMD represents the most frequent inherited muscular dystrophy, affecting nearly one in 3300 male births, and the most common genetic neuromuscular disorder in children. It is caused by recessive mutations in the gene located at Xp21, which codes for the dystrophin protein, and leads to a progressive striated muscles’ weakness and severe physical disability, with loss of ambulation around the age of 12 years [1], [2], [3].

Over time, spinal and chest wall deformities along with impairment of respiratory muscle function lead to hypercapnic respiratory failure, whereas cardiac muscle involvement determines congestive heart failure, with expected death around the second or third decade of life [4], [5], [6].

In addition, most patients experience gastrointestinal and, to a lesser extent, urinary symptoms (i.e. urinary incontinence, hesitancy, straining, weak stream, intermittency, nephrolithiasis, and renal insufficiency) already from a young age. Dysphagia due to swallowing impairment, gastro-esophageal reflux, gastric gas distension, chronic constipation (in up to 46,7% of patients) and diarrhea with possible alternating pattern, blood in stools and fecal incontinence may occur [7], [8], [9]. Also life-threatening complications such as acute gastric dilatation, gastroparesis and intestinal pseudo-obstruction are described, the latter characterized by dilated and fluid-filled small intestine and colon, with possible acute respiratory failure. For this reason, young adults with DMD and a history of abdominal bloating should be routinely investigated at first with abdominal radiography [10].

Autopsy studies have demonstrated edema, fatty infiltration, fragmentation, fibrosis, and waxy degeneration of smooth muscles, resulting in atrophy and thinning of the bowel wall [11,12]. Furthermore, myenteric plexus alterations, with reduced myoelectrical slow wave activity, and reduced availability of nitric oxide, due to lack of dystrophin which acts as an anchor for No-synthase, have been advocated, resulting in an impaired gastro-intestinal motility [13], [14], [15], [16].

Another rare gastrointestinal complication is volvulus, with reported sigmoid localization in patients with a long-standing history of abdominal bloating and constipation along with episodes of pseudo-obstruction and severe bowel wall alterations [10].

However, no cases of transverse colon volvulus were reported in literature till now.

## Conclusion

DMD is associated to many gastrointestinal symptoms, including motility disorders, mostly chronic constipation, and acute complications like pseudo-obstruction and sigmoid volvulus. No mention of transverse colon volvulus is present in the literature, but such a possibility must always be taken into account.

## Patient consent

The patient provided a written informed consent for using anonymized data for publication.
